# Melanoma toolkit for early detection for primary care providers: A pilot study

**DOI:** 10.1111/pcmr.12968

**Published:** 2021-04-23

**Authors:** Victoria E. Orfaly, Elizabeth G. Berry, Elizabeth R. Stoos, Emile Latour, Mirna Becevic, Samantha M. Black, Laura K. Ferris, Alan Geller, Heidi Jacobe, Kelly C. Nelson, Smriti Prasad, Stephanie Savory, Emily H. Smith, Susan M. Swetter, Martin A. Weinstock, Shuai Xu, Sancy A. Leachman

**Affiliations:** ^1^ Department of Dermatology Oregon Health and Science University Portland OR USA; ^2^ Department of Dermatology University of Missouri School of Medicine Columbia MO USA; ^3^ Department of Dermatology University of Texas Southwestern Medical Center Houston TX USA; ^4^ Department of Dermatology University of Pittsburgh Medical Center Pittsburgh PA USA; ^5^ Department of Public Health Practice Harvard T.H. Chan School of Public Health Boston MA USA; ^6^ Department of Dermatology University of Texas Southwestern Medical Center Dallas TX USA; ^7^ Department of Dermatology University of Texas MD Anderson Cancer Center Houston TX USA; ^8^ Department of Dermatology Stanford University Medical Center and Cancer Institute Palo Alto CA USA; ^9^ Department of Dermatology Providence VA Medical Center and Brown University Providence RI USA; ^10^ Department of Dermatology Northwestern University Feinberg School of Medicine Chicago IL USA

## 1 INTRODUCTION

The visible nature of cutaneous melanoma (CM) provides an opportunity to detect and treat suspicious lesions early, thereby potentially reducing mortality. However, one study of 216 patients with melanoma found that only 20% had an established dermatologist whereas 63% received primary care provider (PCP) evaluation within a year prior to diagnosis (Geller et al., 1992). Another study using photographs of lesions found diagnostic accuracy and management of pigmented lesions to be greater by dermatologists than PCPs (Chen et al., 2006). PCPs report that skin cancer early detection is important, but they often lack the appropriate training or time to implement effective skin screenings in their busy practices (Goulart et al., 2011).

Given greater patient access to PCPs than dermatologists, training PCPs to detect and triage concerning pigmented lesions may provide crucial support to the early detection of melanoma. Through Oregon's War on Melanoma public health campaign, we created the “Melanoma Toolkit for Early Detection” (MTED) training curriculum and resource repository for medical providers outside of dermatology. Collaborative efforts from other primary care trainings and an assessment of community needs informed the creation of MTED (Jiang et al., 2017). A novel aspect of MTED is the “Toolkit” design that allows PCPs to choose all or part of a curriculum of evidence‐based training, resources, and patient educational materials. This model supports the adaptability of the MTED intervention, as PCPs can select the materials most meaningful for their practice and capability. During the training development, PCP stakeholders vocalized that they supported this learner‐centered approach as they valued freedom to engage only with the material most relevant to their practice. Educational theory informed both the intervention presentation and the instrument content creation: Mayer's cognitive theory of multimedia learning suggests learning involves actively integrating, attending to, and filtering information (Mayer, 2010). We hypothesized that execution of this learning theory (which involved reducing extraneous processing of irrelevant material, aligning content with learning objectives, and providing goal‐directed practice with feedback) would lead to increased knowledge and confidence in identifying benign and malignant lesions.

## 2 METHODS

The state of Oregon and Oregon Health and Science University (OHSU) has robust PCP networking resources that include the following groups: the Oregon Medical Board, Oregon Communication Health Information Network (OCHIN), and Oregon Rural Practice‐based Research Network (ORPRIN). These groups agreed to recruit participants for this study from primary care networks across the state of Oregon via email and institutional newsletter advertising.

Melanoma toolkit for early detection consists of three self‐paced modules: 1) Screening and Risk Stratification, 2) Visual Perception Training, and 3) the INternet curriculum FOR Melanoma Early Detection (INFORMED) (Choi et al., 2019; Jiang et al., 2017). Each module was self‐paced but expected to take approximately 1 hr for a total of 3 hr for the entire program. Each participant was asked to complete a pre‐ and post‐test consisting of demographic measurements, self‐efficacy (confidence) questions, and image identification questions. A subsequent optional follow‐up test was provided (6 months after training), consisting of a shortened set of images and questions to reassess lesion diagnosis accuracy, self‐efficacy, and practice change. Participants were offered continuing medical education (CME) credit upon completion of both the pre‐ and post‐tests.

## 3 RESULTS

**FIGURE 1:**
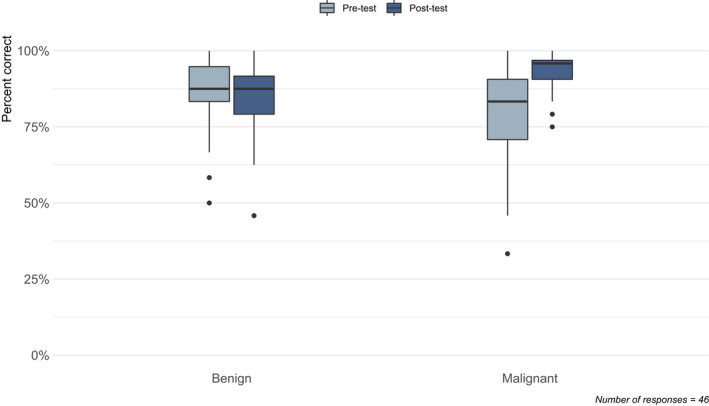
Melanoma toolkit for early detection (MTED) completion was associated with improvement in image classification pre–post test scores. The percentage of pigmented lesions correctly identified as benign or malignant is shown. An average of 6 percentage point improvement was seen overall (*p* < .001), and greater improvement was seen in the ability to identify malignant lesions

Ninety‐six participants engaged in the MTED training and 40 participants completed all modules and tests. Participant demographics are summarized in Table 1. Participants correctly identified 82.9% of lesions as benign or malignant on the pretest and 89.0% on the post‐test. On average, scores increased by 6.0 (95% CI: 3.5 to 8.6) percentage points (*p* < .001, paired *t* test) (Figure 1).

**TABLE 1:** Participant demographics

Demographic (number of total respondents)	*n* (%)
Professional position (84)	Physician 45 (53.6)
	Nurse Practitioner 14 (16.7)
	Other 25 (29.8)
Specialty (80)	Primary care 37 (46.2)
	Dermatology 5 (6.2)
	Emergency Medicine 2 (2.5)
	Other 36 (45)
Years in full‐practice (81)	1–5 years 18 (22.2)
	6–10 years 3 (3.7)
	11–15 years 8 (9.9)
	16–20 years 9 (11.1)
	21+ years 36 (44.4)
	Other 7 (8.6)

**FIGURE 2:**
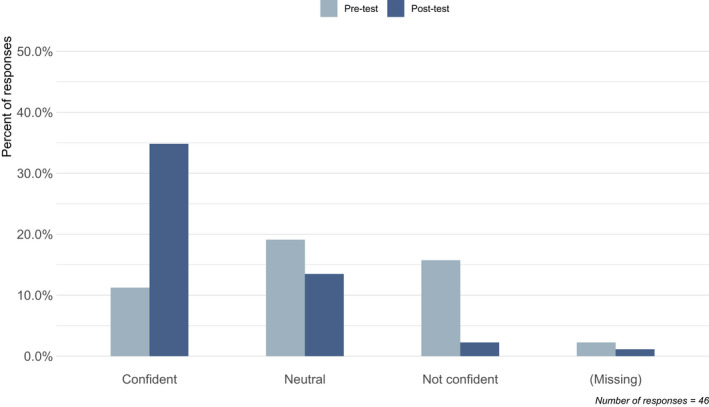
Melanoma toolkit for early detection (MTED) completion was associated with increased melanoma identification confidence. Reported confidence revealed a dramatic increase in “confident” and decrease in “neutral” and “not confident” responses, representing an overall positive shift in confidence following completion of the curriculum

The percent of participants reporting confidence with melanoma identification increased significantly from the pretest (23.3%) to the post‐test (67.4%), an increase of 44.2 (95% CI: 29.3 to 59.0) percentage points (*p* < .001, McNemar's test). Nineteen participants who selected “not confident” with melanoma identification on the pretest selected “confident” on the post‐test (Figure 2).

While the 6‐month follow‐up sample size was limited (*n* = 14), 9 (64.3%) and 8 (57.1%) participants demonstrated long‐term retention of knowledge and confidence, respectively.

## 4 DISCUSSION

In this pilot study, we found that MTED completion by PCPs was associated with increased confidence in diagnosing melanoma and improved ability to correctly identify melanoma. These findings suggest that an online curriculum has the potential to improve early melanoma diagnosis in the primary care setting. However, further work is needed to ensure that this training does not lead to increased PCP confidence without concomitant increased knowledge, especially surrounding when it is appropriate to biopsy a suspicious lesion. The relatively small percentage‐point score improvement in knowledge may have resulted from high baseline test scores, which suggests that a more nuanced test with more challenging lesions may be needed to reflect the knowledge gained. A prior study applying visual perception training for melanoma detection in teaching medical students with lower levels of baseline knowledge demonstrated greater effect in the post‐test with sustained knowledge on repeat testing 4 weeks later (Choi et al., 2019). Our findings suggest similar results.

The primary limitation of this study is its small sample size of participants completing all three modules and tests. Reasons for this are inherent to research with PCPs who have many competing education goals and limited time to pursue them outside their busy clinical practices. This is demonstrated in similar online trainings for PCPs. For example, Samuelson et al. published a self‐paced PTSD course that only had 33% of enrolled participants completed all aspects of the study despite the duration being shorter (2 hr) and a $25 incentive in addition to CME credit (Samuelson et al., 2014). Furthermore, our learner‐centered approach encouraged participants to engage in the modules most meaningful to them rather than completion of all the trainings. This resulted in a self‐selected sample of highly motivated participants who were interested in engaging with the entirety of the curriculum. Future goals include improving follow‐up response to measure provider long‐term retention of knowledge, continued confidence, and lasting behavioral changes. Additionally, we plan to host future iterations of MTED in a learning management system which is capable of collecting more precise data on frequency and length of time participants are engaging with each module. This study shows that MTED has the potential to increase PCP confidence and knowledge, which could lead to improved PCP detection of melanoma.

## References

[pcmr12968-bib-0001] Chen, S. C. , Pennie, M. L. , Kolm, P. , Warshaw, E. M. , Weisberg, E. L. , Brown, K. M. , Ming, M. E. , & Weintraub, W. S. (2006). Diagnosing and managing cutaneous pigmented lesions: Primary care physicians versus dermatologists. Journal of General Internal Medicine, 21(7), 678–682. 10.1111/j.1525-1497.2006.00462.x 16808765 PMC1924688

[pcmr12968-bib-0002] Choi, A. W. , Xu, R. S. , Jacob, S. , Dulmage, B. O. , Colavincenzo, M. L. , Robinson, J. K. , & Xu, S. (2019). Visual perception training: A prospective cohort trial of a novel, technology‐based method to teach melanoma recognition. Postgraduate Medical Journal, 95(1124), 350–352. 10.1136/postgradmedj-2018-136379 31266882

[pcmr12968-bib-0003] Geller, A. C. , Koh, H. K. , Miller, D. R. , Clapp, R. W. , Mercer, M. B. , & Lew, R. A. (1992). Use of health services before the diagnosis of melanoma: Implications for early detection and screening. Journal of General Internal Medicine, 7(2), 154–157. 10.1007/BF02598004 1487762

[pcmr12968-bib-0004] Goulart, J. M. , Quigley, E. A. , Dusza, S. , Jewell, S. T. , Alexander, G. , Asgari, M. M. , Eide, M. J. , Fletcher, S. W. , Geller, A. C. , Marghoob, A. A. , Weinstock, M. A. , & Halpern, A. C. (2011). Skin cancer education for primary care physicians: A systematic review of published evaluated interventions. Journal of General Internal Medicine, 26(9), 1027–1035. 10.1007/s11606-011-1692-y 21472502 PMC3157536

[pcmr12968-bib-0005] Jiang, A. J. , Eide, M. J. , Alexander, G. L. , Altschuler, A. , Asgari, M. M. , Geller, A. C. , Fletcher, S. W. , Halpern, A. C. , & Weinstock, M. A. (2017). Providers' experiences with a melanoma web‐based course: A discussion on barriers and intentions. Journal of Cancer Education: The Official Journal of the American Association for Cancer Education, 32(2), 272–279. 10.1007/s13187-015-0910-4 26391994 PMC4803637

[pcmr12968-bib-0006] Mayer, R. E. (2010). Applying the science of learning to medical education. Medical Education, 44(6), 543–549. 10.1111/j.1365-2923.2010.03624.x 20604850

[pcmr12968-bib-0007] Samuelson, K. W. , Koenig, C. J. , McCamish, N. , Choucroun, G. , Tarasovsky, G. , Bertenthal, D. , & Seal, K. H. (2014). Web‐based PTSD training for primary care providers: A pilot study. Psychological Services, 11(2), 153–161. 10.1037/a0034855 24364595

